# Pyomelanin biosynthetic pathway in pigment-producer strains from the pandemic *Acinetobacter baumannii* IC-5

**DOI:** 10.1590/0074-02760200371

**Published:** 2020-11-06

**Authors:** Érica Fonseca, Fernanda Freitas, Raquel Caldart, Sérgio Morgado, Ana Carolina Vicente

**Affiliations:** 1 Fundação Oswaldo Cruz-Fiocruz, Instituto Oswaldo Cruz, Laboratório de Genética Molecular de Microrganismos, Rio de Janeiro, RJ, Brasil; 2 Universidade Federal de Roraima, Boa Vista, RR, Brasil

**Keywords:** international clone, pyomelanin pigment, adherence, extensively drug resistance, persistence

## Abstract

**BACKGROUND:**

*Acinetobacter baumannii* outbreaks have been associated with pandemic International Clones (ICs), but the virulence factors involved with their pathogenicity are sparsely understood. Pigment production has been linked with bacterial pathogenicity, however, this phenotype is rarely observed in *A. baumannii*.

**OBJECTIVES:**

This study aimed to characterise the reddish-brown pigment produced by *A. baumannii* strains, and to determine its biosynthetic pathway by genomic approaches.

**METHODS:**

Pigment characterisation and antimicrobial susceptibility were conducted by phenotypic tests. The clonal relationship was obtained by pulsed field gel electrophoresis (PFGE) and multi-locus sequence typing (MLST). The genome of an *A. baumannii* was obtained for characterisation of genes involved with pigment production.

**FINDINGS:**

The pyomelanin was the pigment produced by *A. baumannii*. Strains were extensively drug resistant and belonged to the IC-5/ST79. The pyomelanin biosynthetic pathway was determined and presented a particular architecture concerning the peripheral (*tyrB*, *phhB* and *hpd*) and central (*hmgB*, *hmgC* and *hmgR*) metabolic pathway genes. The identification of a distant HmgA homologue, probably without dioxygenase activity, could explain pyomelanin production. Virulence determinants involved with adherence (*csuA/BABCDE* and a T5bSS-carrying genomic island), and iron uptake (*basABCDEFGHIJ*, *bauABCDEF* and *barAB*) were characterised.

**MAIN CONCLUSION:**

There is a biosynthetic pathway compatible with the pyomelanin production observed in persistent *A. baumannii* IC-5 strains.

*Acinetobacter baumannii* is one of the most relevant pathogens associated with nosocomial infections that presents the long-term ability to survive on inanimate surfaces, contributing to national and international clonal dissemination.^1^ The *A. baumannii* outbreaks have been associated with high-risk pandemic lineages, named International Clones (ICs), characterised by a high capacity to persist in clinical environments and by presenting a broad antimicrobial resistance profile.^2^ ^,^ ^3^ However, in spite of *A. baumannii* association with nosocomial and persistent infections, the role of virulence factors in its pathogenesis remains largely obscure. This virulence has been associated with features that enhance its persistence, such as increased adherence, resistance to dissection, biofilm formation, production of capsule and iron uptake.^4^ ^,^ ^5^ ^,^ ^6^

In several bacteria, the production of pigments, as melanins, have been linked with virulence and pathogenicity. Melanins are a black-brown and yellow-red pigments derived from the oxidation of different phenolic compounds.^7^ Depending on the biosynthesis pathway, melanin may be given a different designation, such as pyomelanin, which is a reddish-brown pigment resulted from tyrosine (Tyr) or phenylalanine (Phe) through the accumulation of homogentisic acid (HGA).^8^ This pigment provides protection against oxidative stress and contribute to invasiveness and persistence. Pyomelanin enhances bacterial surface attachment, biofilm formation, extracellular electron transfer, resistance to heavy metals, iron reduction/acquisition, and induces virulence factor expression, which increase the adaptive response to environmental stress.^9^ ^,^ ^10^ The pyomelanin production results from a defect in the catabolism pathway. *Pseudomonas putida* metabolises Phe and Tyr through a peripheral pathway, regulated by the σ^54^-dependent transcriptional activator PhhR, involving hydroxylation of Phe to Tyr by PhhAB, conversion of Tyr into 4-hydroxyphenylpyruvate by TyrB, and formation of HGA by Hpd as the central intermediate. HGA is then catabolised by a central catabolic pathway that involves the homogentisate dioxygenase (HmgA), fumarylacetoacetate hydrolase (HmgB), and maleylacetoacetate isomerase (HmgC), finally yielding fumarate and acetoacetate.^11^ Mutations or deletions that result in loss of HmgA function, as well as overexpression of *hmgR*, a *hmgA* repressor from the Tet^R^ family, lead to an accumulation of HGA.^12^ ^,^ ^13^ The accumulated HGA is then secreted from the cell via the HatABCDE ABC transporter, where it auto-oxidises, and self-polymerises to form pyomelanin.^14^ The production of this pigment is quite common in species such as *Legionella*, *Vibrio cholerae* and *Pseudomonas* sp.^9^ ^,^ ^10^ ^,^ ^11^ ^,^ ^12^ ^,^ ^13^ However, pyomelanin production is a rare phenotype in *A. baumannii*,^15^ and the genes involved with its biosynthesis have not yet been unveiled.

This study reports the occurrence of persistent *A. baumannii* strains producing a brown diffusible pigment resembling the pyomelanin, which caused an outbreak in a hospital of the Amazon Basin, Brazil. Based on whole genome analyses of a representative strain, we unravel the biosynthetic pathway involved with the production of this pigment.

## MATERIALS AND METHODS

*Clinical data, bacterial strains and antimicrobial susceptibility test* - From October, 2016 to April, 2018, 12 *A. baumannii* producing a brown diffusible pigment were recovered from inpatients hospitalised in different wards of the General Hospital of Roraima (HGR), placed in Boa Vista, a city embedded in the Amazon Basin, Brazil (Table). Species identification was determined by the automated VITEK2, and confirmed to be *A. baumannii* by sequencing the 16S rRNA and the *bla*_OXA-51_ genes.

The antimicrobial susceptibility test was determined by assessing the minimal inhibitory concentration (MIC) using E-Test strips (AB BIODISK), according to Clinical and Laboratory Standards Institute (CLSI) guidelines,^16^ for the following antibiotics commonly used for treating *A. baumannii* infections: gentamicin, amikacin, imipenem, meropenem, ciprofloxacin, ampicillin/sulbactam, piperacillin/tazobactam, ceftazidime, cefepime, trimethoprim/sulfamethozaxole, tetracycline and minocycline. The MIC of polymyxin B was assessed by the broth microdilution with antibiotic concentrations ranged from 0.1 μg/mL to 64 μg/mL. The current definition criteria for classifying *A. baumannii* antimicrobial resistance was applied.^17^

**TABLE:** Clinical and genotypic features of the XDR pyomelanogenic *Acinetobacter baumannii* strains

Strains	Isolation date	PFGE	MLST^PAS/OXF^ (IC)	Ward	Clinical specimen
AB4353	Oct/21/16	A	ST79/ST758 (IC-5)	ICU	Catheter tip
AB77	Jan/05/17	A	ST79/ST758 (IC-5)	Others hospital wards	Wound secretion
AB2299	Feb/12/17	A	ST79/ST758 (IC-5)	Emergency	Bronchial aspirate
AB1077	Mar/08/17	A	ST79/ST758 (IC-5)	ICU	Tracheal secretion
AB1113	Mar/08/17	A	ST79/ST758 (IC-5)	ICU	CRL
AB81	Oct/02/17	A1	ST79/ST758 (IC-5)	ICU	Tracheal secretion
AB04-RR5	Jan/01/18	A1	ST79/ST758 (IC-5)	ICU	Blood
AB28-RR5	Jan/17/18	A	ST79/ST758 (IC-5)	ICU	Tracheal secretion
AB41-RR4	Jan/19/18	A	ST79/ST758 (IC-5)	ICU	Tracheal secretion
AB51-RR5	Jan/26/18	A	ST79/ST758 (IC-5)	ICU	Tracheal secretion
AB04-RR6	Apr/25/18	A	ST79/ST758 (IC-5)	ICU	Catheter tip
AB05-RR6	Apr/29/18	A	ST79/ST758 (IC-5)	Others hospital wards	Catheter tip

CRL: cephalorachidian liquid.

*Phenotypic characterisation of brownish pigment produced by A. baumannii strains* - The strains were grown overnight on Mueller-Hinton (MH) and trypticase soy agar (TSA) media plates at different temperatures (28ºC, 35ºC and 40ºC) to verify the influence on pigment production. To investigate whether the pigment is the pyomelanin resulted from the tyrosine metabolism, the 12 pigment-producing *A. baumannii* strains were grown in a minimal medium (T-Medium),^18^ with the tyrosine and glutamate as the sole carbon sources. The pyomelanin-producing *A. baumannii* 456MDp,^15^ kindly provided by Dr Beatriz M Moreira, and the *A. baumannii* ATCC 19606 were also included in this test as positive and negative controls, respectively.

An additional test was performed to determine which tyrosine metabolic pathway was involved with the brown pigment production. Therefore, the effect of sulcotrione [2-(2-chloro-4-methane sulfonylbenzoyl)-1,3-cyclohexanedione)], an inhibitor of tyrosine metabolism via homogentisic acid,^19^ was evaluated by growing the isolates in the T-medium in the presence of different concentrations (2.5, 10, 15 and 20 mM) of sulcotrione.

*Determination of genetic relatedness of A. baumannii strains* - The genetic relationship among the 12 pigment-producing *A. baumannii* strains and between these strains and the pyomelanin-producing *A. baumannii* 456MDp, previously identified in a hospital from Rio de Janeiro,^15^ were assessed by pulsed field gel electrophoresis (PFGE) and multilocus sequence typing (MLST) using both Pasteur (PAS) and Oxford (OXF) schemes (https://pubmlst.org/abaumannii/) available in the *A. baumannii* MLST website.

*Whole genome sequencing and genome annotation* - The genome sequence of one representative pigment-producing strain (AB4353) were obtained with the Illumina HiSeq 2500 sequencer using Nextera XT paired-end run with a ˜500-bp insert library at the High-Throughput Sequencing Platform of the Oswaldo Cruz Foundation (Fiocruz, Rio de Janeiro, Brazil). The quality of the reads was assessed with FASTQC and *de novo* assembling was performed with the SPAdes 3.5 assembler with default settings. Gene prediction and annotation were performed with Rapid Annotations using Subsystems Technology (RAST) server and Prokka software (https://github.com/tseemann/prokka). The resistome was assessed with the Comprehensive Antibiotic Resistance Database (CARD) (https://card.mcmaster.ca/). The mobilome and virulome were assessed with IslandViewer4 (https://www.pathogenomics.sfu.ca/islandviewer/) and VRprofile 2.0 (https://bioinfo-mml.sjtu.edu.cn/VRprofile/) web servers, respectively. AB4353 genome sequence has been submitted to GenBank under accession no. JAAXKU000000000.1.

## RESULTS AND DISCUSSION

*Characterisation of brownish-producer A. baumannii strains* - The 12 clinical *A. baumannii* strains producing a brown diffusible pigment were phenotypically and genotypically characterised. All of them presented the extensively-drug resistant (XDR) phenotype, since they were susceptible only to polymyxin B and tetracyclines. All strains presented the same MIC values as follow: gentamicin (64 µg/mL); amikacin (≥ 256 µg/mL); imipenem (≥ 32 µg/mL); meropenem (≥ 32 µg/mL); ciprofloxacin (≥ 32 µg/mL); piperacillin/tazobactam (128 µg/mL); ceftazidime (64 µg/mL); cefepime (64 µg/mL); trimethoprim/sulfamethoxazole (≥ 32 µg/mL); ampicillin/sulbactam (128 µg/mL); tetracycline (1.5 µg/mL); minocycline (1 µg/mL); polymyxin B (0.125 µg/mL).

**Fig. 1:**
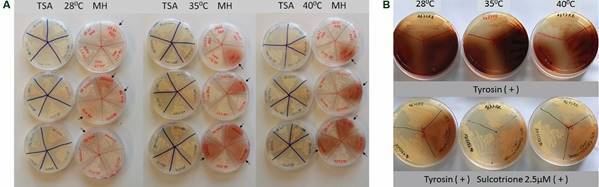
pyomelanin production by the XDR *Acinetobacter baumannii* strains. (A) The conditions and temperatures used in the test are shown. Arrows indicate the pyomelanogenic *A. baumannii* from this study chosen as representative strains for these tests (AB4353, AB1077, AB1113, AB41-RR4). Other *A. baumannii* strains were used as negative controls; (B) production of pyomelanin at minimal T-medium in the presence and in the absence of sulcotrione inhibitor at different temperatures. The image shows the pigment production by two pyomelanogenic *A. baumannii* representative strains of this study (AB4353 and AB77) and by the 456MDp strain used as positive control.

It was verified that the pigment production depends on several growth condition factors to be manifested (temperature, culture medium, growth phase). All strains were able to produce the pigment on MH medium at all tested temperatures after 20 h, with a more prominent production at higher temperatures (35ºC and 40ºC) (Fig. 1A, data shown for AB4353, AB1077, AB1113, AB41-RR4), as previously demonstrated.^15^ ^,^ ^20^ It was verified that production of pigment at higher temperatures was due to the induction of *melA* (*hpd*), which is responsible for the HGA synthesis,^20^ suggesting the role of this mechanism in the adaptive response to environmental stress. On the other hand, no pigment was observed on TSA medium.

Phenotypic tests to verify whether the brown pigment resulted from the tyrosine catabolic pathway revealed that, after 20 h of incubation on T-medium in different temperatures, a brown diffusible pigment was observed in the 12 *A. baumanniii* and in the positive control 456MDp strain (Fig. 1B, data shown for AB4353, AB77 and 456MDp), while no pigment production was observed on the negative control ATCC 19606 (data not shown). Moreover, a significant reduction in the brown pigment production occurred on T-medium plus 2.5 µM of sulcotrione (Fig. 1B). Considering that this substance is an inhibitor of tyrosine metabolism via HGA pathway, and that pyomelanin production results from its accumulation and efflux, it could be inferred that this brownish pigment corresponded to pyomelanin.

*Genetic relatedness and epidemiology of pyomelanin-producer A. baumannii* - The PFGE revealed that the 12 XDR pyomelanin-producing *A. baumannii* strains were clonally related, however, no genetic relationship was observed between these strains and the pyomelanogenic *A. baumannii* 456 MDp strain recovered from a hospital in Rio de Janeiro in 2010.^15^ The MLST revealed that all of them belonged to ST79^PAS^/ ST758^OXF^, which corresponds to the high-risk pandemic International Clone V (IC-5), while the 456MDp strain belonged to ST1079^PAS^ already identified in China in 2015 (MLST metadata). The IC-5 is prevalent in clinical settings spread in Brazil and South America,^21^ however, the pigment production has never been highlighted as a phenotypic trait of this IC neither in Brazil nor in other continents. Interestingly, this lineage has persisted in HGR for more than one year (19 months), which could be resulted from an increased adaptive fitness.

MLST was also performed using both MLST schemes (Oxford and Pasteur) with the complete genome sequence of AB4353 as query, and it was confirmed that this strain belongs to ST79^PAS^ and ST758^OXF^. According to MLST database, there are a remarkable diversity of STs in Oxford scheme that correspond to ST79 in Pasteur scheme. A recent search in this database (September 2020) revealed that ST79 strains circulating in Brazil corresponded to ST233, ST227, ST258, ST1283, ST1284, ST1285, ST1615, ST1935 in Oxford scheme. Interestingly, no ST79^PAS^/ST758^OXF^ was found among the Brazilian strains deposited in MLST database until that occasion. The only entry of ST79^PAS^/ST758^OXF^ corresponded to a strain (AB030) recovered in Canada from a blood infection case. This finding suggests that, although belonging to the IC-5, the lineage ST79^PAS^/ST758^OXF^ is not prevalent in South America.

*The resistome of AB4353 -* Resistome prediction analyses of AB4353 revealed the presence of several genes, conferring resistance to aminoglycosides (*aac(6’)-Ian*, *aac(3’)-IIe*, *strA* and *strB*), chloramphenicol (*cmlA*), sulfonamide (*sul1*), β-lactams (*bla*_TEM-1b_, *bla*_ADC-5_ and *bla*_OXA-65_) and carbapenems (*bla*_OXA-23_), corroborating the observed XDR phenotype. Interestingly, most of these ARGs were in the context of a genomic plasticity region of 48.4 kb that resembles a prophage, and several of them were associated with insertion sequences: IS*91* - *bla*_TEM-1b_ - *aac(3’)-IIe* - IS*Kpn11*; IS*3 - cmlA*; IS*Aba3 - bla*_OXA-23_; IS*Aba1* - *aac(6’)-Ian*. The *strA* and *strB* were found in a putative integrative and conjugative element (ICE) of 108 kb. This ICE also harboured genes involved with metabolic functions and adaptation, such as *umuC*, associated with DNA repair, *teR*, associated with resistance to tellurite and detoxification, and *ars* genes (*arsRDA*), involved with arsenic resistance. No insertion sequence was found in the vicinity of *bla*_ADC-5,_ found in the context of another prophage, and *bla*_OXA-65_, which was embedded in the chromosome.

*Virulome of pyomelanin-producer AB4353 strain: adherence, iron uptake and desiccation tolerance* - Bacterial adherence constitutes an essential step in the colonisation process. *In silico* analysis of AB4353 genome revealed the presence of a 18 kb adherence-related genomic island previously identified in AbH120-A2, an *A. baumannii* strain with a remarkable adherence ability responsible for a large nosocomial outbreak in Spain from 2006 to 2008.^22^ This island carried the Type Vb secretion system from the two-partner System (TPS) family composed by TpsA (AbFhaB) and TpsB (AbFhaC), a large exoprotein involved with Heme utilisation and adhesion and its translocator channel, respectively. This adhesion-related secretion system had already been found in other Gram-negative bacteria, and it is considered one of the main virulence factors in *Bordetella pertussis*.^23^ Interestingly, the AbH120-A2 (2006-2008) from Spain and AB4353 (2017) from Brazil belongs to the IC-5 (ST79), demonstrating the increased adaptive fitness and the remarkable spread potential of this lineage. Such adaptation could be due to the presence of this adhesion-related island, among other factors, which has been probably contributing to IC-5 persistence in clinical settings worldwide for, at least, 10 years. Additionally, other determinants associated with biofilm formation and adherence phenotypes were also identified in AB4353, such as the biofilm-associated protein (Bap) and the CsuA/BABCDE usher-chaperone system.^24^ ^,^ ^25^ ^,^ ^26^

The iron uptake capacity has been considered an important component for bacterial growth and survival under iron-limiting conditions found in host environment, also contributing to pathogenicity. The AB4353 harbour the siderophore Acinetobactin operon identical to that found in the ATCC 19606^T^, composed by *basABCDEFGHIJ, bauABCDEF and barAB* genes*,* involved with biosynthesis, utilisation and siderophore release, respectively.^27^

Desiccation tolerance contributes to the remarkable persistence character of *A. baumannii*, allowing it to become a successful pathogen in the nosocomial environment. The two-component System BfmRS is directly involved with the production of the desiccation resistance phenotype in this species.^28^ Two residues in BfmR, Leu230 and Thr85, are crucial to the BfmR activity and the control of stress responses, which protect *A. baumannii* cells during desiccation. The deduced BfmR from AB4353 presented the canonical residues and is identical to that of profoundly desiccation-tolerant strains,^28^ indicating that AB4353 may have this desiccation tolerance phenotype. In fact, as aforementioned, this strain has persisted in HGR clinical settings for, at least, 19 months. Moreover, it has been shown that the copy number of *umuD* and *umuC* error-prone DNA polymerase V genes may directly contribute to desiccation-induced mutagenesis.^29^ AB4353 presented one copy of *umuD* and three copies of *umuC*, which may be contributing to increase the mutagenesis rates involved with desiccation-tolerant phenotype.

**Fig. 2:**
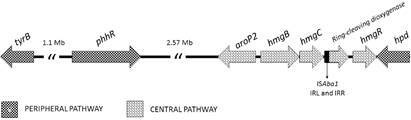
gene organisation of the pyomelanin biosynthetic pathway in *Acinetobacter baumannii* AB4353. Genes are represented by arrows and the central and peripheral pathways genes are highlighted with different patterns.

*Genomic characterisation of the pyomelanin biosynthetic pathway* - Pyomelanin biosynthetic pathway is well known in *Pseudomonas* species.^13^ ^,^ ^14^ ^,^ ^18^ ^,^ ^20^ However, although the pyomelanin production had been demonstrated once in *A. baumannii*,^15^ its biosynthesis remains to be characterised in this species. Thus, we performed comparative genomic analysis to identify and characterise the genes involved with pyomelanin production in AB4353. Homolog genes of *hmgR*, *hmgB* and *hmgC,* involved with pyomelanin central catabolic pathway, were characterised in AB4353, sharing 29%, 45% and 46% deduced amino acid identity with those from *P. putida*, respectively. A homologue of *aroP2* gene, which encodes an aromatic amino acid permease, was found contiguous to the putative *hmgB* in AB4353 (Fig. 2), with a gene arrangement similar to that of *P. putida* KT2440.^11^

The HmgA is a ring-cleaving dioxygenase from the Dioxygenase Superfamily. Although no *hmgA* homologue has been identified in AB4353, a putative gene whose deduced product presented the type I ring-cleaving dioxygenase conserved domain, which is related to the main function of HmgA, was found between *hmgC* and *hmgR* in AB4353 (Fig. 2). Therefore, it suggested the presence of a distant homologue of *hmgA* with no dioxygenase activity in this strain.

The *in silico* analyses revealed that AB4353 harboured the *phhR*, *hpd* and *tyrB* genes from the peripheral pathway (Fig. 2), which presented 42%, 67% and 45% amino acid identity with those from *P. putida*, respectively. As found for some other genera, the *hpd* and *tyrB* were not linked to the *phh* operon in AB4353, as observed in *P. putida*.^11^ In fact, the *phhAB* were absent in AB4353, and the *hpd* was associated with the *hmg* genes (Fig. 2), as in *Pseudomonas syringae*, *Xanthomonas axonopodis*, *Caulobacter crescentus*, *Bradyrhizobium japonicum*, *Mesorhizobium loti*, and *Sinorhizobium meliloti*.^11^ Considering that the conversion of phenylalanine in tyrosine is mediated by *phhAB*, and that these genes are absent in AB4353, it can be assumed that a pathway other than hydroxylation of phenylalanine is probably involved in the tyrosine biosynthesis as demonstrated elsewhere.^11^

Comparison of the peripheral and central pathways of *Pseudomonas* species and other genera demonstrated a high heterogeneity in gene synteny.^11^ In fact, AB4353 displayed a new gene organisation concerning both peripheral and central pathways. Interestingly, blastN analysis revealed a conserved synteny of pyomelanin pathway genes among several *A. baumannii* genomes, including two genomes from IC-5 lineage (AB120-02 and AB421) recovered from outbreaks in Spain in 2006-2008 and 2010.^22^ ^,^ ^30^ The unique difference is that in AB421 the *hmgC* was separated from the putative ring-cleaving dioxygenase gene by a 3.6 kb segment. As aforementioned, the pyomelanin formation depends on the export of the accumulated HGA. AB4353 harboured the entire *hat* ABC transporter gene cluster, sharing 57% (HatA), 69% (HatB), 35% (HatC), 31% (HatD) deduced amino acid identity with those from *P. aeruginosa* UCBPP-PA14.^14^

Therefore, i) the presence of the peripheral pathway genes (*phhR*, *tyrB* and *hpd*) responsible for HGA formation from tyrosine metabolism; ii) the presence of a distant *hmgA* homologue, which is probably not functional, resulting in the HGA cytoplasmic accumulation; and iii) the presence of *hatABCDE* ABC transporter, which pumps HGA, allowing it to self-polymerise into pyomelanin out of the cell, indicate that AB4353 presents the minimum requirements for pyomelanin biosynthesis, and that its production involves a pathway similar to that described in *Pseudomonas* species.^11^ ^,^ ^12^

*In conclusion* - The unraveling of the pyomelanin biosynthetic pathway in *A. baumannii*, involved with the manifestation of a supposed rare phenotype in this species, is indicative that a surveillance of pigment production by clinical *A. baumannii* strains should be considered due to the association of pyomelanin with virulence in bacteria. Indeed, there are several gaps on the mechanisms involved with *A. baumannii* virulence.

## References

[1] Rocha IV, Xavier DE, Almeida KRH, Oliveira SR, Lea NC (2018). Multidrug-resistant Acinetobacter baumannii clones persist on hospital inanimate surfaces. Braz J Infect Dis.

[2] Zarrilli R, Pournaras S, Giannouli M, Tsakris A (2013). Global evolution of multidrug-resistant Acinetobacter baumannii clonal lineages. Int J Antimicrob Agents.

[3] Karah N, Sundsfjord A, Towner K, Samuelsen Ø (2012). Insights into the global molecular epidemiology of carbapenem non-susceptible clones of Acinetobacter baumannii. Drug Resist Updat.

[4] Jones CL, Clancy M, Honnold C, Singh S, Snesrud E, Onmus-Leone F (2015). Fatal outbreak of an emerging clone of extensively drug-resistant Acinetobacter baumannii with enhanced virulence. Clin Infect Dis.

[5] McConnell MJ, Actis L, Pachón J (2013). Acinetobacter baumannii human infections, factors contributing to pathogenesis and animal models. FEMS Microbiol Rev.

[6] Giannouli M, Antunes LC, Marchetti V, Triassi M, Visca P, Zarrilli R (2013). Virulence-related traits of epidemic Acinetobacter baumannii strains belonging to the international clonal lineages I-III and to the emerging genotypes ST25 and ST78. BMC Infect Dis.

[7] Nosanchuk JD, Casadevall A (2003). The contribution of melanin to microbial pathogenesis. Cell Microbiol.

[8] Yabuuchi E, Ohyama A (1972). Characterization of "pyomelanin"-producing strains of Pseudomonas aeruginosa. Int J Syst Bacteriol.

[9] Chatfield CH, Cianciotto NP (2007). The secreted pyomelanin pigment of Legionella pneumophila confers ferric reductase activity. Infect Immun.

[10] Valeru SP, Rompikuntal PK, Ishikawa T, Vaitkevicius K, Sjöling A, Dolganov N (2007). Role of melanin pigment in expression of Vibrio cholerae virulence factors. Infect Immun.

[11] Arias-Barrau E, Olivera ER, Luengo JM, Fernández C, Galán B, García JL (2004). The homogentisate pathway a central catabolic pathway involved in the degradation of L-phenylalanine, L-tyrosine, and 3-hydroxyphenylacetate in Pseudomonas putida. J Bacteriol.

[12] Rodríguez-Rojas A, Mena A, Martín S, Borrell N, Oliver A, Blázquez J (2009). Inactivation of the hmgA gene of Pseudomonas aeruginosa leads to pyomelanin hyperproduction, stress resistance and increased persistence in chronic lung infection. Microbiology.

[13] Ranjan VK, Saha T, Mukherjee S, Chakraborty R (2018). Draft genome sequence of a novel bacterium, Pseudomonas sp strain MR 02, capable of pyomelanin production, isolated from the Mahananda River at Siliguri, West Bengal, India. Genome Announc.

[14] Hunter RC, Newman DK (2010). A putative ABC transporter, hatABCDE, is among molecular determinants of pyomelanin production in Pseudomonas aeruginosa. J Bacteriol.

[15] Coelho-Souza T, Martins N, Maia F, Frases S, Bonelli RR, Riley LW (2014). Pyomelanin production a rare phenotype in Acinetobacter baumannii. J Med Microbiol.

[16] CLSI - Clinical and Laboratory Standards Institute (2020). Performance standards for antimicrobial susceptibility testing: 30th edition CLSI supplement M100.

[17] Magiorakos AP, Srinivasan A, Carey RB, Carmeli Y, Falagas ME, Giske CG (2012). Multidrug-resistant, extensively drug-resistant and pandrug-resistant bacteria an international expert proposal for interim standard definitions for acquired resistance. Clin Microbiol Infect.

[18] Arai T, Hamajima H, Kuwahara S (1980). Pyomelanin production by Pseudomonas aeruginosa I. Transformation of pyomelanin productivity. Microbiol Immunol.

[19] Turick CE, Caccavo F, Tisa LS (2008). Pyomelanin is produced by Shewanella algae BrY and affected by exogenous iron. Can J Microbiol.

[20] Zeng Z, Cai X, Wang P, Guo Y, Liu X, Li B (2017). Biofilm formation and heat stress induce pyomelanin production in deep-sea Pseudoalteromonas sp SM9913. Front Microbiol.

[21] Caldart RV, Fonseca EL, Freitas F, Rocha L, Vicente AC (2019). Acinetobacter baumannii infections in Amazon Region driven by extensively drug resistant international clones, 2016-2018. Mem Inst Oswaldo Cruz.

[22] Pérez A, Merino M, Rumbo-Feal S, Álvarez-Fraga L, Vallejo JA, Beceiro A (2017). The FhaB/FhaC two-partner secretion system is involved in adhesion of Acinetobacter baumannii AbH12O-A2 strain. Virulence.

[23] Melvin JA, Scheller EV, Noël CR, Cotter PA (2015). New insight into filamentous hemagglutinin secretion reveals a role for full-length FhaB in. Bordetella virulence. mBio.

[24] Loehfelm TW, Luke NR, Campagnari AA (2008). Identification and characterization of an Acinetobacter baumannii biofilm-associated protein. J Bacteriol.

[25] Tomaras AP, Dorsey CW, Edelmann RE, Actis LA (2003). Attachment to and biofilm formation on abiotic surfaces by Acinetobacter baumannii involvement of a novel chaperone-usher pili assembly system. Microbiology.

[26] Brossard KA, Campagnari AA (2012). The Acinetobacter baumannii biofilm-associated protein plays a role in adherence to human epithelial cells. Infect Immun.

[27] Mihara K, Tanabe T, Yamakawa Y, Funahashi T, Nakao H, Narimatsu S (2004). Identification and transcriptional organization of a gene cluster involved in biosynthesis and transport of acinetobactin, a siderophore produced by Acinetobacter baumannii ATCC 19606T. Microbiology.

[28] Farrow JM, Wells G, Pesci EC (2018). Desiccation tolerance in Acinetobacter baumannii is mediated by the two-component response regulator BfmR. PLoS One.

[29] Norton MD, Spilkia AJ, Godoy VG (2013). Antibiotic resistance acquired through a DNA damage-inducible response in Acinetobacter baumannii. J Bacteriol.

[30] Merino M, Alvarez-Fraga L, Gómez MJ, Aransay AM, Lavín JL, Chaves F (2014). Complete genome sequence of the multiresistant Acinetobacter baumannii strain AbH12O-A2, isolated during a large outbreak in Spain. Genome Announc.

